# Association between obesity and the risk of skin and soft tissue infections in European populations: A systematic review

**DOI:** 10.1016/j.ijregi.2026.100911

**Published:** 2026-05-06

**Authors:** Bohua Ren, Zeyu Wang, Salman Rawaf, Celine Tabche

**Affiliations:** School of Public Health, Imperial College London, London, United Kingdom

**Keywords:** Obesity, Skin and soft tissue infections, Cellulitis, Hidradenitis suppurativa, Europe, Systematic review

## Abstract

**Background:**

Obesity is highly prevalent in Europe and may increase susceptibility to skin and soft tissue infections (SSTIs), yet European evidence has not been synthesized systematically.

**Objectives:**

To determine whether obesity increases the risk of SSTIs among European adults and summarize related epidemiologic evidence.

**Methods:**

Following Preferred Reporting Items for Systematic reviews and Meta-Analyses 2020 registered CRD420251081476, five databases (PubMed, Scopus, Web of Science, Embase, HMIC) were searched for English-language studies published between 2005 and 2025. Eligible studies included European adults (≥18 years) with obesity, defined as body mass index ≥30 kg/m². Risk of bias was assessed using Newcastle-Ottawa Scale and Joanna Briggs Institute tools.

**Results:**

A total of 20 studies were included. Most reported a positive association between obesity and increased risk of cellulitis, erysipelas, boils/abscesses, postpartum wound infection, or hidradenitis suppurativa. Several studies showed dose–response patterns with increasing body mass index. Hidradenitis suppurativa accounted for a large proportion of the evidence base and was considered separately from classical acute SSTIs.

**Conclusion:**

Obesity is consistently associated with higher risk of several SSTI-related outcomes in European adults. Standardized outcome definitions and prospective studies are needed, alongside trials evaluating whether weight reduction lowers SSTI incidence or recurrence.

## Highlights

• Obesity is consistently linked to a higher risk of skin and soft tissue infections in Europe.
• Strongest associations observed for cellulitis, erysipelas, and abscesses.
• Strong dose–response relationship between body mass index and infection risk.
• Hidradenitis suppurativa shows a consistent obesity association.
• Findings support integrating obesity into skin and soft tissue infections risk assessment.

## Introduction

Obesity is a chronic, complex, and relapsing disease characterized by abnormal or excessive adiposity that impairs health and is associated with substantial morbidity, reduced quality of life, and premature mortality [1]. It is now recognized as one of the most important public health challenges worldwide. According to the World Health Organization (WHO), the global burden of overweight and obesity has risen sharply over recent decades, with billions of adults now affected [1]. The health consequences of obesity are extensive and include increased risks of type 2 diabetes, cardiovascular disease, musculoskeletal disorders, some cancers, and impaired physical functioning [1,2]. In parallel, obesity also imposes major economic costs on health systems and societies, adding further urgency to prevention and management efforts [3].

The WHO European Regional Obesity Report 2022 estimated that almost 60% of adults in the region are living with overweight or obesity, with prevalence continuing to rise across most countries [4]. Upstream drivers, including obesogenic food environments, reduced opportunities for physical activity, socioeconomic inequalities, and wider commercial and structural determinants, have contributed to this sustained increase [5]. As a result, obesity has become a major concern not only for chronic non-communicable disease prevention but also for broader health system resilience and clinical risk management.

Although obesity is most often discussed in relation to cardiometabolic disease, there is growing evidence that it may also increase susceptibility to infection [2]. Reviews of obesity and infectious disease have described obesity as a state of chronic low-grade inflammation, accompanied by altered immune regulation, impaired innate and adaptive immune responses, and metabolic dysfunction, which may reduce host defense [2]. Obesity has also been linked to delayed wound healing, impaired tissue perfusion, and structural changes in the skin and subcutaneous tissues, all of which may plausibly increase the risk of skin and soft tissue infections (SSTIs) [2,6].

SSTIs comprise a diverse group of infections affecting the skin, subcutaneous tissue, fascia, and sometimes muscle, ranging from relatively common and uncomplicated conditions, such as cellulitis, erysipelas, and abscesses, to more severe and potentially life-threatening diseases [7,8]. These infections are clinically important because they are common; frequently recurrent; and associated with considerable use of primary care, emergency, and inpatient services [7,8]. Established risk factors include diabetes, chronic edema, lymphedema, venous insufficiency, skin barrier disruption, smoking, and immunological compromise [7,9,10]. In many cases, these factors co-occur with obesity, making it important to determine whether obesity independently contributes to SSTI risk.

Several biological and local tissue mechanisms support such a relationship. Obesity is associated with chronic systemic inflammation, dysregulated cytokine and adipokine signaling, and impairment of neutrophil and lymphocyte function, which, together, may reduce effective immune responses to cutaneous pathogens [2,5]. At the local level, obesity may alter the skin environment through increased sweating, deeper skin folds, greater mechanical friction, and prolonged moisture retention, thereby promoting maceration, barrier breakdown, and microbial overgrowth [10]. Obesity is also associated with chronic edema and lymphatic dysfunction, particularly, in the lower limbs, which may predispose to recurrent cellulitis and erysipelas by impairing tissue integrity and local immune defense [9]. These pathways may be further compounded by comorbid conditions such as diabetes and vascular disease, which are associated with impaired wound healing and infection susceptibility [2].

Existing evidence increasingly supports an epidemiologic association between obesity and selected SSTI-related outcomes. A recent systematic review and meta-analysis reported a significant association between obesity and cellulitis, whereas broader reviews of obesity and infection have similarly identified skin infections as a key concern [10]. Strong associations have also been reported between obesity and hidradenitis suppurativa (HS), a chronic inflammatory follicular disease that frequently presents with painful nodules, abscesses, drainage, and recurrent suppuration in intertriginous areas [11]. Although HS is not a classical acute bacterial SSTI, it is closely linked to obesity and is frequently discussed in relation to cutaneous infectious burden and skin breakdown. This creates an important conceptual challenge for evidence synthesis, particularly, in reviews that aim to examine obesity and SSTI-related outcomes more broadly.

Despite the clinical and public health relevance of this topic, evidence from European populations has not been synthesized systematically. A regional focus on the WHO European Region is important not only because of its high burden of overweight and obesity but also because obesity-related care pathways, population structures, coding practices, and patterns of SSTI diagnosis and management may differ across health systems.[5]. Restricting the review to this region, therefore, allows more contextually relevant interpretation of the evidence within a defined epidemiologic and policy setting. Existing studies are dispersed across several countries and study designs; use different definitions of obesity; and report heterogeneous outcomes, including cellulitis, abscesses, postoperative wound infection, and HS. In addition, studies differ in the extent to which they adjust for key confounders such as diabetes, smoking, chronic edema, and socioeconomic status, limiting direct comparison and complicating interpretation.

A focused synthesis of European evidence is therefore needed. Clarifying whether obesity is consistently associated with SSTI-related outcomes in adults across the WHO European Region may help improve clinical risk stratification, strengthen prevention efforts, and identify priorities for future research. It may also support a more integrated view of obesity management, in which obesity is considered not only a chronic disease but also a contributor to infection risk and health care burden.

This systematic review, therefore, aimed to synthesize evidence on the association between obesity and SSTI-related outcomes among adults living in countries of the WHO European Region. Secondary objectives were to examine variation by infection subtype, assess study quality, identify key sources of heterogeneity, and summarize implications for clinical practice and public health.

## Methods

This systematic review was conducted in accordance with the Preferred Reporting Items for Systematic reviews and Meta-Analyses 2020 statement [12]. The protocol was registered in the International Prospective Register of Systematic Reviews (CRD420251081476) on June 26, 2025. Ethical approval was not required because the review used only published data.

### Eligibility criteria

This study predefined the eligibility criteria for population, exposure, control, and outcome before data retrieval.

*Population:* Adults aged 18 years or older from countries within the WHO European Region, across community, primary care, or hospital settings.

*Exposure:* Obesity is defined by the original study (typically, body mass index [BMI] ≥30 kg/m²), although studies using clinically recorded obesity, central adiposity measures, or high BMI categories were also eligible.

*Controls/comparator*: Non-obese, lower-BMI, or normal-weight reference groups, as defined by the original study.

*Outcome:* Clinically diagnosed, self-reported, or coded SSTI-related outcomes, including cellulitis, erysipelas, abscesses, boils, gangrene, necrotizing soft tissue infection, wound infection, breast abscess, and HS. Because HS represented a substantial part of the retrieved evidence base and is commonly studied in relation to obesity and cutaneous infection burden, it was retained but analyzed separately from classical acute SSTIs.

We included observational studies published in English in peer-reviewed journals from January 1, 2005 to July 7, 2025. The review was restricted to adults because obesity classification, infection epidemiology, and clinical care pathways differ between pediatric/adolescent and adult populations and combining these groups would have increased heterogeneity and reduced interpretability. Studies in non-English languages were excluded for feasibility reasons; however, this may have introduced language bias and led to the omission of relevant evidence from non–English-speaking countries within the WHO European Region. We excluded pediatric studies, non-European populations, case reports, conference abstracts, reviews, and studies that did not report an obesity-SSTI association or sufficient relevant data.

### Search strategy

We searched PubMed, Scopus, Web of Science, Embase, and HMIC. Embase and HMIC were accessed through Ovid. Searches covered January 1, 2005, to July 7, 2025, and the databases were last searched on July 7, 2025. The search strategy used controlled vocabulary and free-text terms related to obesity, BMI, SSTIs, cellulitis, abscess, erysipelas, HS, and Europe. Reference lists of included articles were also screened (Supplementary Appendix 1).

Gray literature was not included because the review was restricted to peer-reviewed observational studies to maintain consistency in study selection and appraisal. However, exclusion of gray literature may have increased the risk of publication bias and should be considered when interpreting the findings.

### Screening

Records were imported into Covidence, and duplicates were removed automatically and manually. Two reviewers independently screened titles and abstracts, followed by a full-text review of potentially eligible articles. Disagreements were resolved through discussion with a third reviewer.

**Figure 1:**
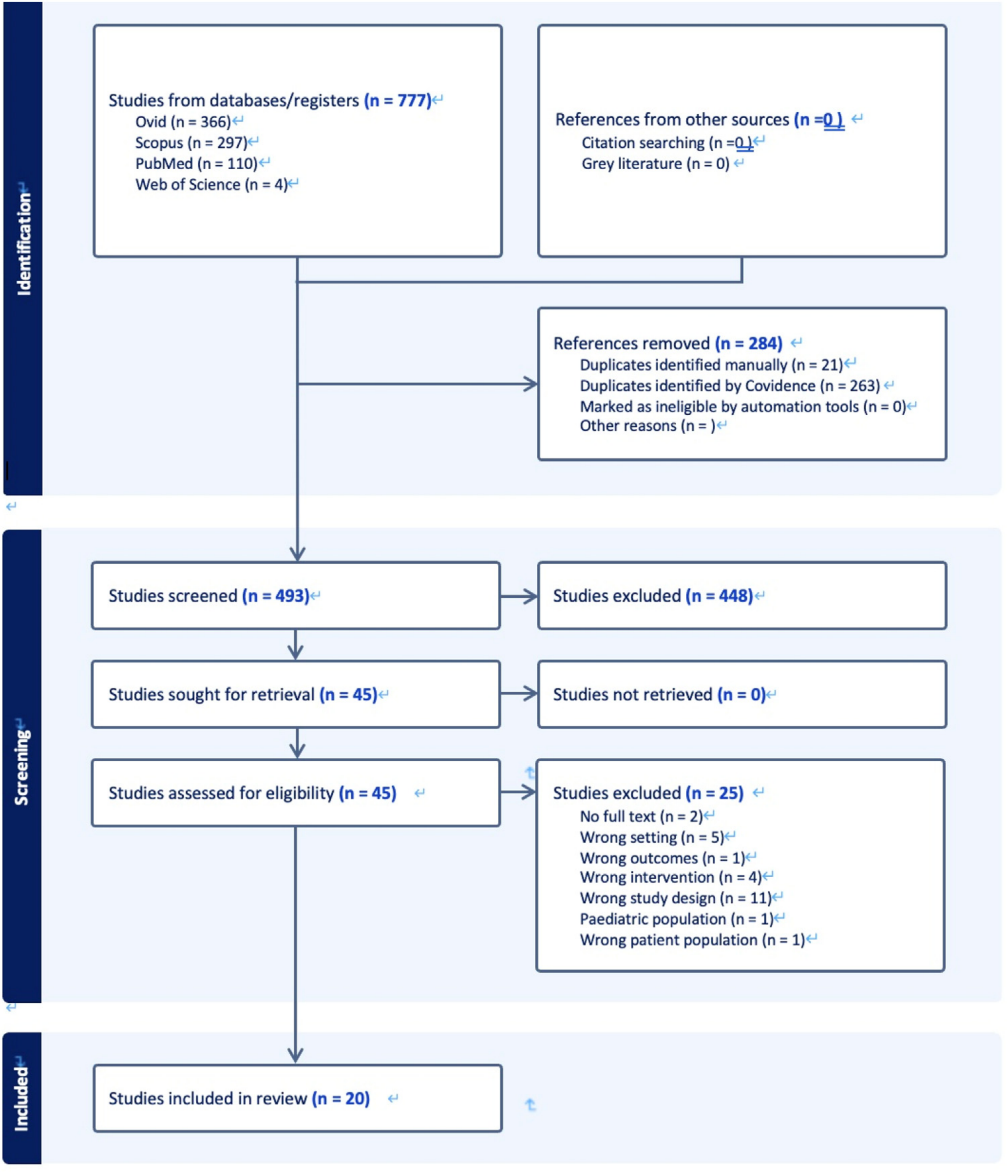
PRISMA flowchart of data selection from Covidence. PRISMA, Preferred Reporting Items for Systematic reviews and Meta-Analyses.

A total of 1012 records were identified: 366 through Ovid (Embase and HMIC), 110 through PubMed, 297 through Scopus, and 239 through Web of Science. After the removal of 284 duplicates, 728 records underwent title and abstract screening. Of these, 683 were excluded, and 45 full-text articles were assessed. A total of 20 studies met the inclusion criteria. A total of 25 full-text articles were excluded for the following reasons: wrong study design (n = 11), wrong setting or region (n = 5), wrong exposure (n = 4), no full text (n = 2), pediatric population (n = 1), wrong outcome (n = 1), and wrong patient population (n = 1). The Preferred Reporting Items for Systematic reviews and Meta-Analyses flow diagram should be submitted separately as Figure 1.

### Data extraction

Data were extracted using a predefined standardized extraction form. One reviewer performed the initial extraction, and a second reviewer checked the extracted data for accuracy and completeness. Any discrepancies or uncertainties were resolved through discussion with the review team. Information extracted included the author, year, country, setting, study design, sample size, population characteristics, obesity definition, SSTI-related outcome definition, effect estimates, adjustment variables, and main findings. Where study data were missing, unclear, or inconsistently reported, this was documented and considered in the narrative synthesis and risk of bias assessment. Where multiple effect estimates were reported, the most fully adjusted estimate was prioritized where available.

### Risks of bias assessment

Cohort and case-control studies were assessed using the Newcastle-Ottawa scale [13]. Analytical cross-sectional studies were assessed using the Joanna Briggs Institute critical appraisal checklist [14]. The Mendelian randomization (MR) studies were appraised narratively, with attention to instrument validity, pleiotropy, and confounding assumptions. Studies were classified overall as low, moderate, or high risk of bias.

### Data synthesis

A meta-analysis was not performed due to substantial heterogeneity in exposure definitions, SSTI outcomes, study designs, and reported effect measures. Findings were therefore synthesized narratively in line with Synthesis Without Meta-analysis guidance [15].

## Results

### Characteristics of included studies

A total of 20 studies published between 2008 and 2024 were included [11,[16], [17], [18], [19], [20], [21], [22], [23], [24], [25], [26], [27], [28], [29], [30], [31], [32], [33], [34]] (Supplementary Table 1). Most were conducted in Nordic countries, with additional studies from the United Kingdom, the Netherlands, Italy, France, Ireland, Serbia, Turkey, and Israel. Turkey and Israel are geographically in Asia but fall within the WHO European Region.

All included studies were observational. The evidence base comprised prospective cohort studies, case-control studies, cross-sectional studies, MR study [18], and one phenome-wide observational analysis [20]. Sample sizes ranged from fewer than 200 participants in some clinical cohorts to more than 800,000 in national data sets. A total of 12 studies focused on HS [11,21,[23], [24], [25],[27], [28], [29],[31], [32], [33], [34]], whereas the remainder examined cellulitis, erysipelas, boils and abscesses, postpartum wound infection, breast abscess, gangrene, chronic lower-limb ulceration, or necrotizing soft tissue infection.

### Exposure and outcome definitions

All studies used obesity or BMI as the primary exposure variable; however, the definitions differ. Most studies used a BMI ≥30 kg/m^2^ as the criterion for obesity. Some studies used more refined BMI categorizations, such as Axelsson *et al.* [17], who classified BMI into overweight (25.0-29.9), obesity class I (30.0-34.9), grade II (35.0-39.9), and morbid obesity (≥40), whereas Revuz *et al.* [11] categorized BMI as normal weight (≤24), overweight (25-29), and obese (≥30). Several prospective studies analyzed BMI as a continuous variable. Butler-Laporte *et al.* [18] found, using genetic tool variable analysis, that for each 1-unit increase in BMI SD (∼4.8 units), the risk of SSTI hospitalization increased by ∼11%. Hyppönen *et al.* [20] reported that a 1-SD increase in BMI was associated with approximately a twofold increase in the risk of superficial cellulitis/abscess. Harpsøe *et al.* [19] also categorized BMI into four grades (<18.5, 18.5-24.9, 25-29.9, ≥30) and assessed risk differences relative to normal weight. Some studies used non-standardized methods, such as Kiralj *et al.* [30], who determined obesity status solely based on medical records.

In HS-related studies, the definition of obesity also varies. Most studies use a BMI ≥30 kg/m² criterion, but Delany *et al*. [31] combined overweight and obesity using a BMI >25 kg/m² criterion [30]. Some studies used specialized measurement methods: Miller *et al.* [32] in Denmark used bioelectrical impedance analysis to measure body fat percentage and compare it between patients with HS and a control group.

In the Netherlands, Vossen *et al.* [34] combined the waist-to-hip ratio to distinguish between “central obesity” and “peripheral obesity'” to explore the impact of fat distribution besides BMI.

Outcome definitions varied markedly. Classical acute SSTIs included cellulitis, erysipelas, superficial abscesses, boils, perianal abscess, gangrene, and necrotizing infection. Procedure-related outcomes included postpartum wound infection and breast abscess. HS-related studies assessed disease prevalence, diagnostic odds, or disease severity.

### Type of SSTI

Evidence for classical acute SSTIs was broadly consistent in showing higher risk with obesity. In the Danish National Birth Cohort, obesity was associated with markedly higher risks of several infections, including cellulitis and erysipelas; the increase was strongest for erysipelas [19]. A Danish cohort of blood donors similarly found that obesity increased overall infection risk and was strongly associated with skin abscesses, with some evidence of stronger effects in men [22].

In the UK primary care, obesity was associated with increased risk of boils and abscesses [26]. A Swedish population-based study also reported higher risk of perianal abscess recurrence among patients with obesity [16]. In the UK Biobank phenome-wide analysis, higher BMI was associated with substantially increased odds of superficial cellulitis or abscess, chronic lower-limb ulcers, and gangrene [20]. The MR study by Butler-Laporte *et al.* [18] found that elevated genetically predicted BMI was associated with higher risk of hospital admission for infections, including SSTIs.

Not all studies were fully concordant. A Serbian necrotizing fasciitis cohort reported obesity as a common comorbidity, but the small sample and descriptive design limited inference [30].

Procedure-related outcomes also suggested an adverse association with obesity, although the pattern differed by outcome. In a large Swedish postpartum cohort, higher maternal BMI was associated with increased risk of postpartum wound infection after delivery, whereas the association with breast abscess did not follow the same pattern and appeared weaker or inverse [17]. This was one of the few exceptions to the overall direction of association.

HS accounted for most of the included studies. Across this literature, obesity was consistently associated with higher odds of HS and, in several studies, with greater disease severity.

Revuz *et al.* [11] reported that obesity was strongly associated with HS in French case-control data. Ingram *et al.* [33] found obesity to be independently associated with HS in a UK population-based study using the Clinical Practice Research Datalink. Lapi *et al.* [25] similarly identified obesity as an independent correlate of HS in Italian primary care, whereas Shalom *et al*. [27] reported a stronger obesity-HS association in individuals younger than 50 years.

Other studies documented the high burden of overweight and obesity among patients with HS. Kromann *et al*. [24] observed greater HS prevalence and severity with increasing body weight. Delany *et al*. [31] reported that most patients in an Irish HS cohort were overweight or obese, and Theut Riis *et al*. [28] found that Danish blood donors with self-reported HS had higher mean BMI than those without HS. Kjærsgaard Andersen *et al*. [23] showed that increasing BMI predicted progression to more severe HS over time, whereas Yüksel and Basım [29] found higher BMI associated with severe disease in a Turkish clinical sample.

A few HS-related studies used alternative adiposity measures. Vossen *et al*. [34] assessed central and peripheral obesity patterns, and Miller *et al*. [32] examined body composition using bioelectrical impedance analysis. Overall, the HS literature showed a strong and consistent association with obesity, but these findings should be interpreted separately from acute bacterial SSTIs because HS is a chronic inflammatory condition rather than a classic infection.

### Dose-response relationship

Several studies demonstrated a dose–response relationship. In the Danish cohort of women, infection risk increased across BMI categories [19]. The Swedish postpartum study showed a rising risk of wound infection with increasing maternal BMI [17]. In UK Biobank, each SD increase in BMI was associated with higher odds of multiple skin-related outcomes [20]. Revuz *et al*. [11] also observed increasing HS risk with rising BMI.

Some subgroup differences were reported. Kaspersen *et al*. [22] found stronger obesity-associated infection risk in men than in women for some outcomes. Shalom *et al*. [27] observed a stronger obesity–HS association in younger adults. However, these subgroup findings were not available consistently across all studies.

#### Risk of bias

A total of 15 studies were judged to be at low risk of bias and five at moderate risk; none was judged high risk (Table 1). The overall quality was strengthened by the inclusion of large population-based cohorts and two MR analyses. The principal limitations were heterogeneity in exposure and outcome definitions, variation in adjustment for confounders, and reliance in some studies on routine coding or self-reported measures.

**Table 1:** Quality assessment summary for the 20 included studies.

Study type	Total study	Low risk/high quality	Moderate risk/quality	High risk/low quality
Cross-sectional (NOS)	8	5	3	0
Cohort (NOS)	6	6	0	0
Case-control (NOS)	3	2	1	0
Observational (JBI)	3	2	1	0
Total	20	15	5	0

Abbreviations: JBI, Joanna Briggs Institute; NOS, Newcastle–Ottawa Scale.

## Discussion

This systematic review found that obesity is consistently associated with an increased risk of several SSTI-related outcomes among adults across the WHO European Region. Although the magnitude of association varied by study design, population, and outcome definition, the overall direction of effect was highly consistent. The clearest evidence was observed for cellulitis, erysipelas, boils and abscesses, and selected hospital-based skin infection outcomes. A substantial proportion of the included literature also demonstrated a strong association between obesity and HS. Across studies, individuals with obesity generally had a higher risk of SSTIs than those of normal weight, with effect estimates ranging from modest increases to several-fold higher risks.

Several studies demonstrated a dose–response relationship, whereby increasing BMI was associated with progressively higher infection risk [11,17,19,20]. This gradient strengthens the plausibility of the association and suggests that the relationship is not limited to a binary comparison between obese and non-obese groups.

The inclusion of MR analyses provides additional support for a potential causal contribution of higher BMI to infection risk. Studies using genetically predicted BMI reported increased risks of infection-related outcomes, including SSTIs [18,20]. Although MR approaches are subject to methodological assumptions, these findings are consistent with observational evidence and reduce the likelihood that the association is entirely explained by residual confounding.

HS accounted for a substantial proportion of the included studies and showed a strong and consistent association with obesity across multiple European settings [11,25,27,33]. Several studies also reported increasing disease severity with higher BMI [23,24,29]. However, HS should be interpreted as a chronic inflammatory condition with suppurative features rather than a classic acute bacterial SSTI. Its inclusion broadens the scope of the review but requires careful interpretation when comparing findings across infection subtypes.

Variation in the strength of association was observed across different SSTI outcomes. Obesity appeared to be particularly strongly associated with lower-limb infections, such as cellulitis and erysipelas, which may reflect the contribution of chronic edema, venous insufficiency, and lymphatic dysfunction in these conditions [9,35]. In contrast, associations with certain procedure-related infections, such as breast abscess, were less consistent, with some studies reporting weaker or inverse relationships [17]. These findings highlight the importance of clinical context and underlying pathophysiology when interpreting outcome-specific associations.

Some evidence suggested that the association between obesity and SSTI risk may vary across population subgroups, although findings were not consistent across all studies. For example, cohort data indicated that obesity-related infection risk may be more pronounced in men than in women for certain outcomes [22], whereas other studies suggested stronger associations in younger populations [27]. These differences may reflect variations in fat distribution, comorbidity profiles, and health-seeking behaviors, although the included studies were not designed to examine these mechanisms in detail.

Obesity frequently coexists with other established risk factors for SSTIs, including diabetes, smoking, and chronic edema, which may contribute to infection susceptibility even when statistical adjustment is applied [36]. The persistence of associations after adjustment in several studies suggests that obesity may independently contribute to infection risk while interacting with other clinical and behavioral factors.

The mechanisms underlying this relationship are likely to be multifactorial. Obesity has been associated with chronic low-grade inflammation, altered immune responses, and impaired wound healing, which may reduce host defense against cutaneous pathogens [2]. Local tissue factors, including increased skin folds, moisture retention, and mechanical friction, may further promote barrier disruption and microbial colonization [6]. In addition, obesity-related lymphatic dysfunction and chronic edema, particularly, in the lower limbs, are recognized contributors to recurrent cellulitis and erysipelas [9,35]. Although these mechanisms are biologically plausible, they were not directly evaluated in the included studies.

Findings from international studies outside the WHO European Region provide additional contextual support for these observations. For example, a large cohort study in Korea reported that higher BMI was associated with increased incidence and hospitalization risk for cellulitis, independent of metabolic status [37]. Similarly, a systematic review and meta-analysis from the United States identified a significant association between obesity and cellulitis risk [38]. Other population-based studies have also highlighted variation in risk across ethnic groups and settings [39]. Although these studies were not included in the primary evidence base of this review, their findings are broadly consistent with the European literature and suggest that the association between obesity and SSTIs may represent a general epidemiologic pattern rather than a region-specific phenomenon.

This review has several limitations. First, substantial heterogeneity in exposure definitions, outcome ascertainment, and effect measures precluded quantitative synthesis and limited comparability across studies. Second, although HS was analyzed separately, its inclusion broadens the interpretation of SSTI-related outcomes. Third, most studies were conducted in a limited number of European countries, particularly, Nordic settings and the United Kingdom, which may affect generalizability across the wider region. Fourth, restriction to English-language, peer-reviewed studies introduces the possibility of language bias and publication bias and may have led to omission of relevant evidence from non–English-speaking countries within the WHO European Region. Finally, reliance on routinely collected or self-reported data in several studies may have resulted in misclassification of obesity and infection outcomes.

Despite these limitations, the findings have important clinical and public health implications. The consistency of the association suggests that obesity may be considered in SSTI risk assessment, particularly, among individuals with recurrent infections or relevant comorbidities. In clinical practice, this may support more proactive assessment of skin integrity and early management of local risk factors. However, caution is required when translating these findings into intervention strategies. Current evidence does not establish whether weight reduction directly reduces SSTI incidence or recurrence, and further prospective and interventional research is needed.

Future research should prioritize standardized definitions of SSTIs, consistent adjustment for key confounders, and multicenter prospective studies across diverse European populations. In addition, interventional studies are needed to determine whether weight management and integrated care approaches can reduce the burden of SSTIs. Further work should also continue to distinguish the role of obesity in acute bacterial infections from its role in chronic inflammatory conditions such as HS.

### Policy recommendations

These findings suggest that obesity may be considered in the clinical assessment of SSTI risk, particularly, in individuals with recurrent infections or relevant comorbidities. In primary care and community settings, this may support greater attention to skin integrity, including routine examination and early management of skin breakdown. At a system level, the results highlight the potential value of integrating infection prevention into broader obesity and chronic disease management strategies, although further evidence is needed to determine whether weight reduction directly reduces SSTI incidence or recurrence.

## Conclusion

This systematic review of 20 European studies found that obesity was consistently associated with higher risk of several SSTI-related outcomes, particularly, cellulitis, erysipelas, boils, abscesses, postpartum wound infection, and HS. Evidence from large observational data sets and MR analyses supported the likelihood that higher BMI contributes to increased infection risk, although heterogeneity in outcome definitions and confounder adjustment remains important. Obesity should, therefore, be considered in clinical assessment of SSTI risk, whereas future research should prioritize standardized outcome definitions, broader European prospective cohorts, and interventional studies assessing whether weight reduction lowers SSTI incidence or recurrence.

## Declaration of competing interest

The authors have no competing interests to declare.

## Appendix Supplementary materials

Image, application 1

Image, application 2

Image, application 3
