# Supplementary material for: Association between obesity and the risk of skin and soft tissue infections in European populations: A systematic review
Source: IJID Reg. 2026 May 6;19:100911. doi: 10.1016/j.ijregi.2026.100911 (PMC13224352; doi:10.1016/j.ijregi.2026.100911)
Supplement: Supplementary file 1 [file mmc1.docx]

Appendix 1: Search strategy

| **Database** | **Search strategy** |
| --- | --- |
| PubMed (110) | ("Obesity"[MeSH] OR "obesity"[tiab] OR "obese"[tiab] OR "overweight"[MeSH] OR "overweight"[tiab] OR "body mass index"[tiab] OR "BMI"[tiab] OR "adiposity"[tiab] OR "waist circumference"[tiab] OR "central obesity"[tiab] OR "weight status"[tiab]) AND("Skin Diseases, Bacterial"[MeSH] OR "Soft Tissue Infections"[MeSH] OR "skin and soft tissue infection"[tiab] OR "SSTI"[tiab] OR "cellulitis"[tiab] OR "abscess"[tiab] OR "erysipelas"[tiab] OR "impetigo"[tiab] OR "furuncle"[tiab] OR "carbuncle"[tiab]) AND "Europe"[MeSH] OR "Europe"[tiab] OR "European Union"[tiab] OR "United Kingdom"[tiab] OR "UK"[tiab] OR "Germany"[tiab] OR "France"[tiab] OR "Italy"[tiab] OR "Spain"[tiab] OR "Sweden"[tiab] OR "Denmark"[tiab] OR "Finland"[tiab] OR "Poland"[tiab] OR "Austria"[tiab] OR "Netherlands"[tiab] OR "Belgium"[tiab] OR "Ireland"[tiab] OR "Portugal"[tiab] OR "Greece"[tiab] OR "Norway"[tiab] OR "Switzerland"[tiab] OR "Albania"[tiab] OR "Andorra"[tiab] OR "Armenia"[tiab] OR "Azerbaijan"[tiab] OR "Belarus"[tiab] OR "Bosnia and Herzegovina"[tiab] OR "Bulgaria"[tiab] OR "Croatia"[tiab] OR "Cyprus"[tiab] OR "Czechia"[tiab] OR "Estonia"[tiab] OR "Georgia"[tiab] OR "Hungary"[tiab] OR "Iceland"[tiab] OR "Israel"[tiab] OR "Kazakhstan"[tiab] OR "Kyrgyzstan"[tiab] OR "Latvia"[tiab] OR "Lithuania"[tiab] OR "Luxembourg"[tiab] OR "Malta"[tiab] OR "Monaco"[tiab] OR "Montenegro"[tiab] OR "North Macedonia"[tiab] OR "Republic of Moldova"[tiab] OR "Romania"[tiab] OR "Russia"[tiab] OR "San Marino"[tiab] OR "Serbia"[tiab] OR "Slovakia"[tiab] OR "Slovenia"[tiab] OR "Tajikistan"[tiab] OR "Turkey"[tiab] OR "Turkmenistan"[tiab] OR "Ukraine"[tiab] OR "Uzbekistan"[tiab] AND("Adult"[MeSH] OR "adult"[tiab] OR "adults"[tiab] OR "adult population"[tiab] OR "European population"[tiab]) AND (english[lang]) AND ("2005/01/01"[Date - Publication] : "3000"[Date - Publication]) |
| Ovid (366) | (exp Obesity/ OR obesity.tw. OR obese.tw. OR "body mass index".tw. OR BMI.tw. OR exp Overweight/ OR overweight.tw. OR "waist circumference".tw. OR "central obesity".tw. OR adiposity.tw. OR "weight status".tw.)  AND (exp Skin Diseases, Bacterial/ OR exp Soft Tissue Infections/ OR "skin and soft tissue infection*".tw. OR SSTI.tw. OR cellulitis.tw. OR abscess.tw. OR erysipelas.tw. OR impetigo.tw. OR furuncle.tw. OR carbuncle.tw.)  AND (exp Europe/ OR Europe.tw. OR "European Union".tw. OR "United Kingdom".tw. OR UK.tw. OR Germany.tw. OR France.tw. OR Italy.tw. OR Spain.tw. OR Sweden.tw. OR Denmark.tw. OR Finland.tw. OR Poland.tw. OR Austria.tw. OR Netherlands.tw. OR Belgium.tw. OR Ireland.tw. OR Portugal.tw. OR Greece.tw. OR Norway.tw. OR Switzerland.tw. OR Albania.tw. OR Andorra.tw. OR Armenia.tw. OR Azerbaijan.tw. OR Belarus.tw. OR "Bosnia and Herzegovina".tw. OR Bulgaria.tw. OR Croatia.tw. OR Cyprus.tw. OR Czechia.tw. OR Estonia.tw. OR Georgia.tw. OR Hungary.tw. OR Iceland.tw. OR Israel.tw. OR Kazakhstan.tw. OR Kyrgyzstan.tw. OR Latvia.tw. OR Lithuania.tw. OR Luxembourg.tw. OR Malta.tw. OR Monaco.tw. OR Montenegro.tw. OR "North Macedonia".tw. OR "Republic of Moldova".tw. OR Romania.tw. OR Russia.tw. OR "San Marino".tw. OR Serbia.tw. OR Slovakia.tw. OR Slovenia.tw. OR Tajikistan.tw. OR Turkey.tw. OR Turkmenistan.tw. OR Ukraine.tw. OR Uzbekistan.tw.)  AND (exp Adult/ OR adult.tw. OR adults.tw. OR "adult population".tw. OR "European population".tw.)  Limit to Publication Year: 2005 – Current |
| Scopus (297) | ( TITLE-ABS-KEY ( "obesity" ) OR TITLE-ABS-KEY ( "obese" ) OR TITLE-ABS-KEY ( "overweight" ) OR TITLE-ABS-KEY ( "body mass index" ) OR TITLE-ABS-KEY ( "BMI" ) OR TITLE-ABS-KEY ( "adiposity" ) OR TITLE-ABS-KEY ( "waist circumference" ) OR TITLE-ABS-KEY ( "central obesity" ) OR TITLE-ABS-KEY ( "weight status" ) ) AND ( TITLE-ABS-KEY ( "skin and soft tissue infection" ) OR TITLE-ABS-KEY ( "SSTI" ) OR TITLE-ABS-KEY ( "cellulitis" ) OR TITLE-ABS-KEY ( "abscess" ) OR TITLE-ABS-KEY ( "erysipelas" ) OR TITLE-ABS-KEY ( "impetigo" ) OR TITLE-ABS-KEY ( "furuncle" ) OR TITLE-ABS-KEY ( "carbuncle" ) ) AND ( TITLE-ABS-KEY ( "European Union" ) OR TITLE-ABS-KEY ( "Europe" ) OR TITLE-ABS-KEY ( "United Kingdom" ) OR TITLE-ABS-KEY ( "UK" ) OR TITLE-ABS-KEY ( "Germany" ) OR TITLE-ABS-KEY ( "France" ) OR TITLE-ABS-KEY ( "Italy" ) OR TITLE-ABS-KEY ( "Spain" ) OR TITLE-ABS-KEY ( "Sweden" ) OR TITLE-ABS-KEY ( "Denmark" ) OR TITLE-ABS-KEY ( "Finland" ) OR TITLE-ABS-KEY ( "Poland" ) OR TITLE-ABS-KEY ( "Austria" ) OR TITLE-ABS-KEY ( "Netherlands" ) OR TITLE-ABS-KEY ( "Belgium" ) OR TITLE-ABS-KEY ( "Ireland" ) OR TITLE-ABS-KEY ( "Portugal" ) OR TITLE-ABS-KEY ( "Greece" ) OR TITLE-ABS-KEY ( "Norway" ) OR TITLE-ABS-KEY ( "Switzerland" ) OR TITLE-ABS-KEY ( "Albania" ) OR TITLE-ABS-KEY ( "Andorra" ) OR TITLE-ABS-KEY ( "Armenia" ) OR TITLE-ABS-KEY ( "Azerbaijan" ) OR TITLE-ABS-KEY ( "Belarus" ) OR TITLE-ABS-KEY ( "Bosnia and Herzegovina" ) OR TITLE-ABS-KEY ( "Bulgaria" ) OR TITLE-ABS-KEY ( "Croatia" ) OR TITLE-ABS-KEY ( "Cyprus" ) OR TITLE-ABS-KEY ( "Czechia" ) OR TITLE-ABS-KEY ( "Estonia" ) OR TITLE-ABS-KEY ( "Georgia" ) OR TITLE-ABS-KEY ( "Hungary" ) OR TITLE-ABS-KEY ( "Iceland" ) OR TITLE-ABS-KEY ( "Israel" ) OR TITLE-ABS-KEY ( "Kazakhstan" ) OR TITLE-ABS-KEY ( "Kyrgyzstan" ) OR TITLE-ABS-KEY ( "Latvia" ) OR TITLE-ABS-KEY ( "Lithuania" ) OR TITLE-ABS-KEY ( "Luxembourg" ) OR TITLE-ABS-KEY ( "Malta" ) OR TITLE-ABS-KEY ( "Monaco" ) OR TITLE-ABS-KEY ( "Montenegro" ) OR TITLE-ABS-KEY ( "North Macedonia" ) OR TITLE-ABS-KEY ( "Republic of Moldova" ) OR TITLE-ABS-KEY ( "Romania" ) OR TITLE-ABS-KEY ( "Russia" ) OR TITLE-ABS-KEY ( "San Marino" ) OR TITLE-ABS-KEY ( "Serbia" ) OR TITLE-ABS-KEY ( "Slovakia" ) OR TITLE-ABS-KEY ( "Slovenia" ) OR TITLE-ABS-KEY ( "Tajikistan" ) OR TITLE-ABS-KEY ( "Turkey" ) OR TITLE-ABS-KEY ( "Turkmenistan" ) OR TITLE-ABS-KEY ( "Ukraine" ) OR TITLE-ABS-KEY ( "Uzbekistan" ) ) AND ( TITLE-ABS-KEY ( "adult" ) OR TITLE-ABS-KEY ( "adults" ) OR TITLE-ABS-KEY ( "adult population" ) OR TITLE-ABS-KEY ( "European population" ) ) AND PUBYEAR > 2004 AND ( LIMIT-TO ( LANGUAGE , "English" ) ) |
| Web of Science (4) | TS=("obesity" OR "obese" OR "overweight" OR "body mass index" OR "BMI" OR "adiposity" OR "waist circumference" OR "central obesity" OR "weight status") AND TS=("skin and soft tissue infection" OR "SSTI" OR "cellulitis" OR "abscess" OR "erysipelas" OR "impetigo" OR "furuncle" OR "carbuncle") AND TS=("Europe" OR "European Union" OR "United Kingdom" OR "UK" OR "Germany" OR "France" OR "Italy" OR "Spain" OR "Sweden" OR "Denmark" OR "Finland" OR "Poland" OR "Austria" OR "Netherlands" OR "Belgium" OR "Ireland" OR "Portugal" OR "Greece" OR "Norway" OR "Switzerland" OR "Albania" OR "Andorra" OR "Armenia" OR "Azerbaijan" OR "Belarus" OR "Bosnia and Herzegovina" OR "Bulgaria" OR "Croatia" OR "Cyprus" OR "Czechia" OR "Estonia" OR "Georgia" OR "Hungary" OR "Iceland" OR "Israel" OR "Kazakhstan" OR "Kyrgyzstan" OR "Latvia" OR "Lithuania" OR "Luxembourg" OR "Malta" OR "Monaco" OR "Montenegro" OR "North Macedonia" OR "Republic of Moldova" OR "Romania" OR "Russian Federation" OR "San Marino" OR "Serbia" OR "Slovakia" OR "Slovenia" OR "Tajikistan" OR "Türkiye" OR "Turkmenistan" OR "Ukraine" OR "Uzbekistan") AND TS=("adult" OR "adults" OR "adult population" OR "European population") |
