# Supplementary material for: Association between obesity and the risk of skin and soft tissue infections in European populations: A systematic review
Source: IJID Reg. 2026 May 6;19:100911. doi: 10.1016/j.ijregi.2026.100911 (PMC13224352; doi:10.1016/j.ijregi.2026.100911)
Supplement: Supplementary file 2 [file mmc2.docx]

Appendix 2: Quality assessment

Cross-sectional study (NEWCASTLE - OTTAWA QUALITY ASSESSMENT SCALE)

| Author | Year | Selection Bias Assessment  (Maximum 4 stars) | | | | | | | | Comparability  (Maximum 2 stars) | | Outcome (Maximum 3 stars) | | | | Total score  (Maximum 9 stars) |
| --- | --- | --- | --- | --- | --- | --- | --- | --- | --- | --- | --- | --- | --- | --- | --- | --- |
|  |  | Representativeness of the sample | | Sample size | | Non-respondents | | Ascertainment of the exposure (risk factor) | | Confounding factors are controlled | | Assessment of the outcome | | Statistical Test | |  |
|  |  | selection | score | selection | score | selection | score | selection | score | selection | score | selection | score | selection | score |  |
| Janse et al. | 2016 | Yes/1 | | Yes/1 | | Yes/1 | | Yes/1 | | Yes/2 | | Yes/1 | | Yes/1 | | 8 |
| Kromann et al | 2014 | Yes/1 | | Yes/1 | | No/0 | | Yes/1 | | Yes/2 | | Yes/1 | | Yes/1 | | 7 |
| Shalom et al | 2015 | Yes/1 | | Yes/1 | | No/0 | | Yes/1 | | Yes/2 | | Yes/1 | | Yes/1 | | 7 |
| Riis et al | 2019 | Yes/1 | | Yes/1 | | No/0 | | Yes/1 | | Yes/2 | | Yes/1 | | Yes/1 | | 7 |
| Yüksel & Basım | 2019 | Yes/1 | | Yes/1 | | No/0 | | Yes/1 | | Yes/1 | | Yes/1 | | Yes/1 | | 6 |
| Delany E, et al. | 2017 | Yes/1 | | Yes/1 | | Yes/1 | | Yes/1 | | No/0 | | Yes/1 | | Yes/1 | | 6 |
| Miller et al | 2015 | Yes/1 | | Yes/1 | | Yes/1 | | Yes/1 | | Yes/1 | | Yes/1 | | Yes/1 | | 7 |
| Vossen et al | 2016 | Yes/1 | | Yes/1 | | No/0 | | Yes/1 | | Yes/1 | | Yes/1 | | Yes/1 | | 6 |

Cohort study (NEWCASTLE - OTTAWA QUALITY ASSESSMENT SCALE)

| Author | Year | Selection Bias Assessment (Maximum 4 stars) | | | | | | | | Comparability (Maximum 2 Stars) | | Outcome (Maximum 3 stars) | | | | | | Total score (Maximum 9 stars) |
| --- | --- | --- | --- | --- | --- | --- | --- | --- | --- | --- | --- | --- | --- | --- | --- | --- | --- | --- |
|  |  | Representativeness of the exposed cohort | | Selection of the non-exposed cohort | | Ascertainment of exposure | | Demonstration that the outcome of interest was not present at the start of the study | | Comparability of cohorts based on the design or analysis | | Assessment of the outcome | | Was the follow-up long enough for outcomes to occur | | Adequacy of follow up of cohorts | |  |
|  |  | selection | score | selection | score | selection | score | selection | score | selection | score | selection | score | selection | score | selection | score |  |
| Adamo et al. | 2016 | Yes/1 | | Yes/1 | | Yes/1 | | Yes/1 | | Yes/1 | | Yes/1 | | Yes/1 | | Yes/1 | | 8 |
| Harpsøe MC et al. | 2016 | Yes/1 | | Yes/1 | | Yes/1 | | Yes/1 | | Yes/2 | | Yes/1 | | Yes/1 | | Yes/1 | | 9 |
| Kaspersen et al | 2015 | Yes/1 | | Yes/1 | | Yes/1 | | Yes/1 | | Yes/2 | | Yes/1 | | No/0 | | Yes/1 | | 8 |
| Andersen et al | 2024 | Yes/1 | | Yes/1 | | Yes/1 | | Yes/1 | | Yes/2 | | Yes/1 | | Yes/1 | | Yes/1 | | 9 |
| Lapi et al | 2024 | Yes/1 | | Yes/1 | | Yes/1 | | Yes/1 | | Yes/2 | | Yes/1 | | Yes/1 | | Yes/1 | | 9 |
| Shallcross et al. | 2015 | Yes/1 | | Yes/1 | | Yes/1 | | Yes/1 | | Yes/2 | | Yes/1 | | Yes/1 | | Yes/1 | | 9 |

Case control study (NEWCASTLE - OTTAWA QUALITY ASSESSMENT SCALE)

| Author | Year | Selection Bias Assessment  (Maximum 4 stars) | | | | | | | | Comparability  (Maximum 2 stars) | | Outcome (Maximum 3 stars) | | | | | |  |
| --- | --- | --- | --- | --- | --- | --- | --- | --- | --- | --- | --- | --- | --- | --- | --- | --- | --- | --- |
|  |  | Is the case definition adequate? | | Representativeness of the cases | | Selection of controls | | Definition of controls | | Comparability of cases and controls on the basis of the design or analysis | | Assessment of the exposure | | Same method of ascertainment for cases and controls | | Non-response rate | | Total score  (Maximum 9 stars) |
|  |  | selection | score | selection | score | selection | score | selection | score | selection | score | selection | score | selection | score | selection | score |  |
| Hyppönen et al | 2019 | Yes/1 | | No/0 | | Yes/1 | | Yes/1 | | Yes/1 | | Yes/1 | | Yes/1 | | No/0 | | 6 |
| Revuz et al | 2008 | Yes/1 | | Yes/1 | | Yes/1 | | Yes/1 | | Yes/1 | | Yes/1 | | Yes/1 | | No/0 | | 7 |
| Ingram et al. | 2018 | Yes/1 | | Yes/1 | | Yes/1 | | Yes/1 | | Yes/2 | | Yes/1 | | Yes/1 | | Yes/1 | | 9 |

Descriptive and observational study (JBI Checklist)

|  | **Author (Year)** | **1. Clear inclusion criteria?** | **2. Detailed settings and subjects?** | **3. Valid and reliable exposure measurement?** | **4. Objective measurement of condition?** | **5. Confounding factors identified?** | **6. Confounding strategies stated?** | **7. Valid and reliable outcome measurement?** | **8. Appropriate statistical analysis?** | **Overall Risk of Bias** |
| --- | --- | --- | --- | --- | --- | --- | --- | --- | --- | --- |
|  |  | Yes/No/Unclear/Not applicable | Yes/No/Unclear/Not applicable | Yes/No/Unclear/Not applicable | Yes/No/Unclear/Not applicable | Yes/No/Unclear/Not applicable | Yes/No/Unclear/Not applicable | Yes/No/Unclear/Not applicable | Yes/No/Unclear/Not applicable | Low/Moderate/High |
| 1 | Axelsson et al., 2018 | Yes | Yes | Yes | Yes | Yes | Yes | Yes | Yes | Low |
| 2 | Butler-Laporte et al., 2021 | Yes | Yes | Yes | Yes | Yes | Yes | Yes | Yes | Low |
| 3 | Kiralj et al., 2015 | Yes | Yes | Unclear | Yes | Yes | No | Unclear | Unclear | Moderate |

Risk of bias:
Low risk: ≥ 75% of the items are Yes
Moderate risk: 50% - 74% is Yes, or part of Unclear/No
HIgh risk: < 50% is Yes, or there are key areas No
