# Supplementary material for: Association between obesity and the risk of skin and soft tissue infections in European populations: A systematic review
Source: IJID Reg. 2026 May 6;19:100911. doi: 10.1016/j.ijregi.2026.100911 (PMC13224352; doi:10.1016/j.ijregi.2026.100911)
Supplement: Supplementary file 3 [file mmc3.docx]

**Supplementary Table 1. Descriptive summary of the study characteristics from data extracted from the 20 studies.**

| **Ref** | **Author (year)** | **Study design (country)** | **Sample size** | **Population type** | **SSTI type** | **Obesity definition** | **Findings** |
| --- | --- | --- | --- | --- | --- | --- | --- |
| 34 | Adamo *et al.*  (2016) | Retrospective cohort study  (Sweden) | 18,877 patients (11,138 males, 4557 females) | The Swedish population (all patients treated for perianal abscesses in Sweden between 1997 and 2009) | Perianal abscess | BMI ≥30 kg/m² defined as obesity | Ratio between the prevalence of obesity in the perianal abscess group and the prevalence of the same in the Swedish population (RR)=1.59 (1.45–1.73) |
| 35 | Axelsson *et al.*  (2018) | Observational study  (Sweden) | 841,780 women | The Swedish population of women who gave birth between 2005 and 2012 | Postpartum infection: WI and BA | BMI classification: Overweight (25.0-29.9), obesity class I (30.0-34.9), obesity class II (35.0-39.9), obesity class III (≥40.0) | BMI is associated with an increased risk of postpartum infection in a dose-dependent manner  The risk of infection is increased by 50% in the morbidly obese group(BMI ≥40)  The risk of WI increases with rising BMI  The risk of BA decreases with increasing BMI |
| 36 | Butler-Laporte *et al.* (2021) | Mendelian randomization study  (United Kingdom) | 681,265 participants (BMI GWAS);  UK Biobank cohort >500,000 participants (infectious disease GWAS) | European ancestry population (GIANT Consortium + UK Biobank) | Broadly defined SSTIs | BMI of UKB participants: 27.4 ± 4.8 | An increase in BMI is associated with a higher hospital admission rate for SSTI (OR 1.11, 95% CI 1.09, 1.12). |
| 12 | Harpsøe MC *et al.*, (2016) | Prospective cohort study  (Denmark) | 75,001 women | Women who participated in the Danish National Birth Cohort between 1996 and 2002 (median age 30.7 years) | Cellulitis and abscess, erysipelas, pilonidal cyst, other skin and subcutaneous tissue–related infections | BMI classification: Underweight (<18.5),  normal weight  (referent)(18.5 to <25),  overweight(25 to <30),  obese(≥30) | Dose–response relationship: Risk of skin infections showed a dose–response relationship with increasing BMI  Specific hazard ratios:  Overweight women (BMI 25-<30): HR = 1.27 (95% CI: 1.11-1.46)  Women with obesity (BMI ≥30): HR = 1.78 (95% CI: 1.50-2.10)  Risk for specific SSTI types:  Erysipelas: Highest risk in women with obesity HR = 5.19 (95% CI: 3.38-7.95),over fivefold increased risk  Cellulitis and abscess: Overweight HR = 1.29, Obese HR = 1.64  Other skin infections: Women with obesity HR = 2.16 (95% CI: 1.20-3.89),over twofold increased risk |
| 37 | Hyppönen *et al.* (2019) | Case-control PheWAS  (United Kingdom) | 337,536 participants | UK Biobank participants aged 37-73 years, unrelated white British individuals | Superficial cellulitis and abscess，chronic ulcers of leg and foot，gangrene，inflammatory or dermatological conditions | BMI ≥30 kg/m² defined as obesity | Skin and soft tissue infection outcomes:  Superficial cellulitis and abscess: OR = 2.00 (95% CI: 1.72-2.23) per 1 SD BMI increase, doubled risk.  Chronic ulcers of leg and foot: OR = 3.37 (95% CI: 2.17-5.25) per 1 SD BMI increase over threefold increased risk.  Gangrene: OR = 4.99 (95% CI: 2.54-9.82) per 1 SD BMI increase-nearly fivefold increased risk.  All associations demonstrated strong statistical significance (*P* <5.4×10⁻⁵) |
| 38 | Janse *et al.* (2016) | Cross-sectional observational study  (Netherlands) | 1260 patients with IBD | IBD patients:  Crohn’s disease, n = 634; ulcerative colitis, n = 626 | Hidradenitis Suppurativa | Using BMI as an indicator but without clearly defining the specific BMI threshold for obesity. | The BMI was significantly higher in the HS group (*P* = 0.030)  In the multivariate logistic regression analysis, a higher BMI also appeared to be associated with the occurrence of HS (OR = 1.075) |
| 23 | Kaspersen *et al.* (2015) | Prospective cohort study  (Denmark) | 37,808 healthy blood donors | Healthy blood donors (ages 18-67 years) | Skin and subcutaneous tissue infections，abscesses | BMI ≥30 kg/m² defined as obesity | Abscesses  Women: Obese group HR ~2.5, 150% increased risk  Men: Obese group HR ~3.0, 200% increased risk  Skin and subcutaneous tissue infections - Significant gender difference  Women: No significant association, CI crosses 1.0  Men: Clear association, obese group HR ~2.7, 170% increased risk |
| 39 | Andersen *et al.* (2024) | Prospective cohort study  (Denmark) | 335 patients | Hurley III stage HS patients who have not received biological agent therapy | Hidradenitis Suppurativa | BMI >25 defined as obesity | Each BMI point above 25 at baseline increases the risk of transition to severe hidradenitis suppurativa (HR 1.06) |
| 40 | Kromann *et al.* (2014) | Retrospective questionnaire-based study  (Denmark) | 249 valid respondents (of 383 patients who underwent bariatric surgery) | Patients with obesity who underwent weight loss surgery (gastric bypass or gastric banding surgery at Hvidovre Hospital between 2010 and 2011) | Hidradenitis Suppurativa | BMI >30 kg/m² (Pre-operative BMI range of study subjects: 30.9-70.9 kg/m²) | The prevalence of HS in individuals with obesity is 18.1% (45/249) [95% CI: 18.1 ± 4.8%], which is 5-18 times higher than in the general population. |
| 41 | Lapi *et al.* (2024) | Case-control study  (Italy) | 4,530 HS cases matched to 35,135 controls | Italian primary care patients, aged ≥18 years | Hidradenitis Suppurativa | BMI ≥30 kg/m² defined as obesity | Obesity is a significant risk factor for HS:  Overall analysis: OR = 1.36 (95% CI: 1.18–1.56)  “Confirmed” case analysis: OR = 2.14 (95% CI: 1.43–3.22) |
| 42 | Revuz *et al.* (2008) | Case-control study  (France) | Prevalence survey: n = 10,000 (actual 6887 people)  Case-control study1 (population-based):  Self-reported patients: n = 67  Control group: n = 200  Case-control study2(clinically based):  Medically assessed patients: n = 302  Control group: n = 906 | General population: Representative sample of the French population aged 15 years and older  Clinical population: Consecutive patients with HS who were treated at research centers between 1998 and 2006 | Hidradenitis Suppurativa | Normal weight: BMI ≤24 kg/m²  Overweight: BMI 25-29 kg/m²  Obese: BMI ≥30 kg/m² | For every 1-unit increase in BMI, the risk of HS increases by 12%.  Patients with obesity (BMI ≥30) have a 4.4 times higher risk of developing HS than individuals of normal weight. |
| 43 | Shallcross *et al.*  (2015) | Retrospective cohort study  (United Kingdom) | Total cohort:  n = 164,461，Follow-up cohort: n = 122,473，Male: n = 51,986, Female: n = 70,487 | UK primary care patients (6% of UK population)  All ages, follow-up: 1995-2011 | Boils and Abscesses | BMI ≥30 kg/m² defined as obesity | Obesity (relative risk = 1.3, 95% CI = 1.2 - 1.3) |
| 44 | Shalom *et al.*  (2015) | Cross-sectional study  (Israel) | HS patients: n = 3207, Controls: n = 6412,  Total: n = 9619 | Community-based population from CHS database, covers 52% of Israeli population (4.2 million people), HS diagnosed by dermatologists in primary care centers | Hidradenitis Suppurativa | Obesity: BMI >30 kg/m² | Obesity–HS association: Univariate analysis: Obesity: OR = 1.74 (95% CI: 1.56-1.94), *P* <0.001, Obesity rate: HS 22.2% vs Controls 14.1%.  Multivariate analysis (adjusted for age, sex, smoking): Obesity: OR = 1.71 (95% CI: 1.53-1.91), *P* <0.001,  Obesity increases HS risk by 71%. Stronger association in young patients (≤50 years): Obesity: OR = 2.03 (95% CI: 1.77-2.34), *P* <0.001, Obesity rate: HS 18.5% vs Controls 10%. |
| 45 | Riis *et al.* (2019) | Cross-sectional study  (Denmark) | 27,765 blood donors | Blood donor cohort | Hidradenitis Suppurativa | The average BMI of HS blood donors was 27.0 kg/m², while the average BMI of non-HS blood donors was 25.7 kg/m². | The average BMI of patients with S was 1.3 kg/m² higher, suggesting an association between HS and higher BMI. |
| 46 | Yüksel & Basım (2019) | Retrospective cross-sectional study  (Turkey) | 208 patients | Dermatology and general surgery outpatients | Hidradenitis Suppurativa | Normal weight: 18.5-25 kg/m², Overweight: 25-29 kg/m², Obese: ≥30 kg/m² | Compared with patients with low BMI, patients with high BMI had an 8.9 times higher risk of progressing to severe disease. |
| 47 | Kiralj *et al.* (2015) | Retrospective study  (Serbia) | 216 patients | Surgical inpatients | Necrotizing fasciitis | Obesity as a preexisting disease factor, with no clearly defined BMI cut-off point | Obesity is the second most common preexisting risk factor (17.59%), after drug abuse (18.05%). |
| 48 | Delany *et al.* (2018) | Cross-sectional epidemiological study  (Ireland) | 150 patients | Adult HS patients (aged ≥18 years) attending dermatology outpatient clinics at four Irish hospitals. | Hidradenitis suppurativa | BMI ≥25 kg/m² (overweight or obese) | 81.8% of HS patients are overweight or obese (BMI ≥25 kg/m²)  The average BMI is 32.3 ± 8.6 kg/m²  Female patients have a higher BMI: 33.1 ± 9.2 kg/m² vs. 30.3 ± 6.6 kg/m² in males |
| 49 | Miller *et al.* (2016) | Cross-sectional study  (Denmark) | Hospital HS: n = 32,  population HS: n = 430,  controls: n = 20,780 | Hospital group: Adult patients (aged >18 years) with moderate to severe HS receiving systemic or laser treatment  Population group: HS patients identified through the GESUS study | Hidradenitis suppurativa | Use BIA to assess body composition, including fat percentage, visceral fat, BMI, and other indicators. | Age- and sex-adjusted analyses showed a 10.12% (*P* <0.0001) significantly higher fat percentage in the hospital-based HS group and 3.11% (*P* <0.0001) significantly higher fat percentage for the population-based HS group than controls. |
| 50 | Vossen *et al.*  (2017) | Cross-sectional study  (Netherlands) | HS: n = 106; Controls: n = 212 | Adult dermatology outpatients, mainly Caucasian, age ≥18 years; controls matched for age and sex | Hidradenitis suppurativa (chronic inflammatory skin disease, broad SSTI) | BMI ≥30 kg/m² (WHO); Central obesity: BMI ≥30 + WHR ≥0.90 (men) or ≥0.85 (women); Peripheral obesity: BMI ≥30 + WHR <0.90 (men) or <0.85 (women) | HS patients had significantly higher BMI vs controls (27.8 ± 5.4 vs 25.6 ± 4.8, *P* <0.001). WHR not significantly different. Among those with obesity, HS more often had peripheral fat distribution (43% vs 19%, *P* = 0.036). HS associated with higher BMI but not central obesity by WHR; peripheral fat may promote mechanical friction in HS sites. |
| 51 | Ingram *et al.*  (2018) | Observational and case-control study  (United Kingdom) | CPRD data set: 4,364,308 patients; HS diagnosed: 23,353; additional algorithm-identified proxy cases validated: 10,146 confirmed HS cases; 18,417 possible cases | General population in UK primary care with research-standard CPRD records; adults of all ages | Hidradenitis suppurativa | BMI >30 kg/m² defined as obesity | Prevalence of HS: 0.77% (strict validated definition), up to 1.19% including possible cases. Obesity strongly associated with HS (OR 3.29, 95% CI 3.14-3.45). Other associations: current smoking (OR 3.61), type 2 diabetes (OR 3.39), Crohn’s disease (OR 2.65). HS more common in women (female:male ratio ~2.9:1) and peaked in the 5th decade of life. |
